# 基于室温溶液悬浮法共价有机骨架的制备及其对茶叶中拟除虫菊酯固相微萃取的应用

**DOI:** 10.3724/SP.J.1123.2020.12012

**Published:** 2021-04-08

**Authors:** Qidong YU, Lan ZHANG, Wenmin ZHANG, Jiangfan YANG

**Affiliations:** 1.福建农林大学园艺学院, 福建 福州 350002; 1. College of Horticulture, Fujian Agriculture and Forestry University, Fuzhou 350002, China; 2.福州大学, 食品安全与生物分析教育部重点实验室, 福建 福州 350116; 2. Ministry ofEducation Key Laboratory of Analytical Science of Food Safety and Biology, Fuzhou University, Fuzhou 350116,China; 3.闽江师范高等专科学校, 化学与生物工程系, 福建 福州 350108; 3. Division of Chemical and Biological Engineering, Minjiang Teachers College, Fuzhou 350108, China

**Keywords:** 气相色谱-串联质谱, 固相微萃取, 共价有机骨架材料, 拟除虫菊酯, 茶叶, gas chromatography-tandem mass spectrometry (GC-MS/MS), solid phase microextraction (SPME), covalent organic frameworks (COFs), pyrethroids (PYs), tea

## Abstract

拟除虫菊酯(PYs)类农药被广泛用于茶园病虫害的防治。随着国内外对进出口茶叶中农药残留限量的要求日益严格,我国迫切地需要开发能应用于检测茶叶中痕量PYs的方法。研究通过简单、温和的溶液悬浮法(SSA),在室温下制备了共价有机骨架(COF)材料TpBD,该材料具有优异的热/化学稳定性、较高的孔隙度和比表面积。通过简单物理黏附的方法,将TpBD制备成固相微萃取(SPME)纤维。将准备好的纤维应用于固相微萃取技术并与气相色谱-串联质谱(GC-MS/MS)联用,建立用于检测PYs的高灵敏分析方法。该方法对氟氯氰菊酯、氯氰菊酯、氟氰菊酯、氰戊菊酯和溴氰菊酯的富集倍数为702~2687,并表现出低的检出限(0.1~0.5 ng/L)、宽的线性范围(0.2~800 ng/L)、良好的线性关系(相关系数≥0.9991)、可接受的重复性(RSD≤11.0%, *n*=3)。将所建立的方法用于实际绿茶和乌龙茶样品中PYs残留量的检测,并成功地从实际茶样中检测出5种痕量PYs,并且具有满意的回收率(80.2%~109.5%)。实验结果表明,所建立的分析方法适用于茶叶中PYs农药残留量的分析和检测。此外,通过SSA成功地制备了TpBD材料,证实了该方法具有较好的普适性,具有简便合成其他COFs材料的潜力。

## Abstract (en)

Pyrethroids (PYs) have been widely used to control pests and prevent diseases in tea gardens. However, with the increasingly stringent pesticide testing standards in the import and export trade of tea, there is an urgent need for methods to detect trace amounts of PYs in tea. In this study, a covalent organic framework (COF) material TpBD with excellent thermal/chemical stability, high porosity, and a large specific surface area was prepared by a room-temperature solution-suspension approach (SSA). TpBD-coated solid phase microextraction (SPME) fibers were fabricated by coating the material on etched stainless-steel fibers by a simple physical coating method. The fibers were used in combination with gas chromatography-tandem mass spectrometry (GC-MS/MS) to establish a highly sensitive method for the detection of PYs. The enrichment factors of this method for cyfluthrin, cypermethrin, flucythrinate, fenvalerate, and deltamethrin were 702-2687. The method showed low LODs (0.1-0.5 ng/L), wide linear ranges (0.2-800 ng/L), good linearities (correlation coefficients (*R*)≥0.9991) and acceptable repeatabilities (RSD≤11.0%, *n*=3). Green tea and oolong tea samples were analyzed using the developed method, and trace levels of the five PYs were successfully detected. The recoveries of the spiked PYs in the real green tea and oolong tea samples were in the range of 80.2%-109.5%. Experimental results showed that the established analytical method is suitable for the determination of PY pesticides in tea. Furthermore, the TpBD material was successfully prepared by the SSA method, demonstrating that the method has good universality and excellent potential for the simple synthesis of other COF materials.

我国茶叶生产体量位居世界第一,但在国际市场上缺乏竞争优势,其中一个主要原因是在茶树的种植栽培过程中化学农药,特别是拟除虫菊酯(pyrethroids, PYs)类农药的滥用^[1]^。PYs会在人体内累积,扰乱生物的神经系统和内分泌系统^[2]^,因此,欧盟、美国、日本等组织和国家对进口茶叶制定了越来越严苛的农药检测标准,这对中国茶叶的出口贸易考验极大^[3]^。因此,建立茶叶中PYs检测的新方法对促进我国茶业发展极其重要^[4,5]^。

固相微萃取技术(SPME)作为一种绿色的样品前处理技术已被用于对PYs的预富集^[6]^。其中,涂层材料是SPME方法的关键因素,直接影响建立方法的选择性、灵敏度和重现性。当前,各种纳米材料例如金属氧化物^[7]^、碳氮化合物^[8]^、金属有机骨架材料(MOF)^[9]^等已经被探索作为新型SPME涂层。近年来,共价有机骨架(COFs)材料因具有高比表面积、良好的热/化学稳定性、易于合成后修饰等优点,而成为优异的吸附剂^[10,11]^。PYs是一类分子直径在1.6 nm左右的疏水性芳香族化合物^[12]^,而*β*-酮烯胺COF(TpBD)具有芳香环和疏水性的大型二维网状拓扑结构^[13,14]^。考虑到PYs和COFs之间存在*π-π*相互作用和疏水效应,TpBD可能对PYs具有很好的吸附效果^[15]^。然而,TpBD的制备常依赖复杂苛刻的反应条件,例如高温、高压以及冷冻脱气等^[16,17,18]^。苛刻的合成条件限制了TpBD在SPME上的应用。因此,如何发展简便的方法合成TpBD,并应用在SPME方法上引起分析领域的重视。

本实验提出通过室温溶液悬浮法(SSA)合成具有良好结晶度、高孔隙度和优异稳定性的TpBD。在室温条件下,反应溶液悬浮静置3 d合成TpBD。将TpBD制成SPME涂层纤维,与气相色谱-串联质谱(GC-MS/MS)联用,经过实验条件的优化,建立了一种适用于茶树种植栽培过程中常用的5种PYs的检测方法,并考察了该方法的检出限、回收率和重复性等。最后,将所建立的分析方法用于乌龙茶和绿茶实际样品中PYs的检测。

## 1 实验部分

### 1.1 仪器与试剂

TRACE 1300/TSQ 8000 Evo气相色谱-串联质谱联用仪、EscaLab 250Xi X-射线光电子能谱仪、Nicolet 6700傅里叶变换红外光谱仪(美国Thermo Fisher Scientific公司); Tecnai G2 F20透射电子显微镜(美国FEI公司); JSM-6300F扫描电子显微镜(日本JEOL公司); ASAP2020物理吸附仪(上海Micromeritics公司); DIL402C热重分析仪(德国Netzsch公司); X' Pert Pro MPD X射线粉末衍射仪(荷兰Panalytical公司)。

1,4-二氧六环(dioxane,纯度99.5%)、乙酸(纯度99.8%)以及标准品溴氰菊酯(deltamethrin, DEL)、氯氰菊酯(cypermethrin, CYP)、氟氰菊酯(flucythrinate, FLU)、氰戊菊酯(fenvalerate, FEN)和氟氯氰菊酯(cyfluthrin, CYF)(100 μg/mL)均购自中国Aladdin试剂有限公司;三醛基间苯三酚(Tp,纯度98%)和联苯胺(BD,纯度98%)购自吉林中科研伸科技有限公司;乙腈(ACN,纯度99.5%)、甲醇(MeOH,纯度99.5%)、四氢呋喃(THF,纯度99.5%)、二氯甲烷(DCM,纯度99.5%)、丙酮(CP,纯度99.5%)和乙醇(EtOH,纯度99.5%)购自中国国药试剂有限公司;不锈钢纤维(SSF)购自当地的超市。实验用水均为Milli-Q净水器所制得的超纯水(18.2 MΩ·cm)。

用丙酮对拟除虫菊酯类农药标准品进行逐级稀释,配制成一系列不同浓度的标准工作溶液,供后续实验使用。

### 1.2 分析条件

色谱柱:TG-5MS石英毛细管柱(30 m×0.2 mm×0.25 μm);进样口温度:280 ℃;载气:高纯He(纯度>99.999%);流速:1.0 mL/min;不分流进样;升温程序:70 ℃保持1 min,以25 ℃/min的速率升至250 ℃,保持19 min。

离子源:EI源;电离能量:70 eV;接口温度:300 ℃;四极杆温度:150 ℃;离子源温度:230 ℃;采用赛默飞TSQ 8000 Evo三重四极杆质谱仪特有的定时-选择反应检测扫描(Timed-SRM)模式,对实验数据进行采集。5种PYs的其他质谱参数见表1。

**表 1:** 5种拟除虫菊酯类农药的保留时间、定性离子和定量离子

Compound	Retention time/min	Qualitative ions (*m/z*)	Quantitative ion (*m/z*)
Cyfluthrin (CYF)	17.49	206, 199, 226	206
Cypermethrin (CYP)	17.81	181, 152, 180	181
Flucythrinate (FLU)	19.35	199, 157, 451	199
Fenvalerate (FEN)	21.45	225, 167, 419	225
Deltamethrin (DEL)	25.45	253, 172, 181	253

### 1.3 TpBD材料及其涂层纤维的制备

将已经报道^[19]^的室温溶液悬浮合成亚胺类COF(COF-LZU1)的方法进行改进后用于合成TpBD(见图1a)。将42.0 mg Tp、56.0 mg BD和2.0 mL的1,4-二氧六环加入10 mL离心管中,超声至单体完全溶解。取350 μL 3 mol/L乙酸水溶液加入混合物中,超声混匀后密封离心管,静置于室温下3 d,然后离心5 min (5000 r/min)收集沉淀物,并分别用THF、DCM和CP清洗至溶液呈无色,最后在真空条件下60 ℃干燥24 h。

**图 1:**
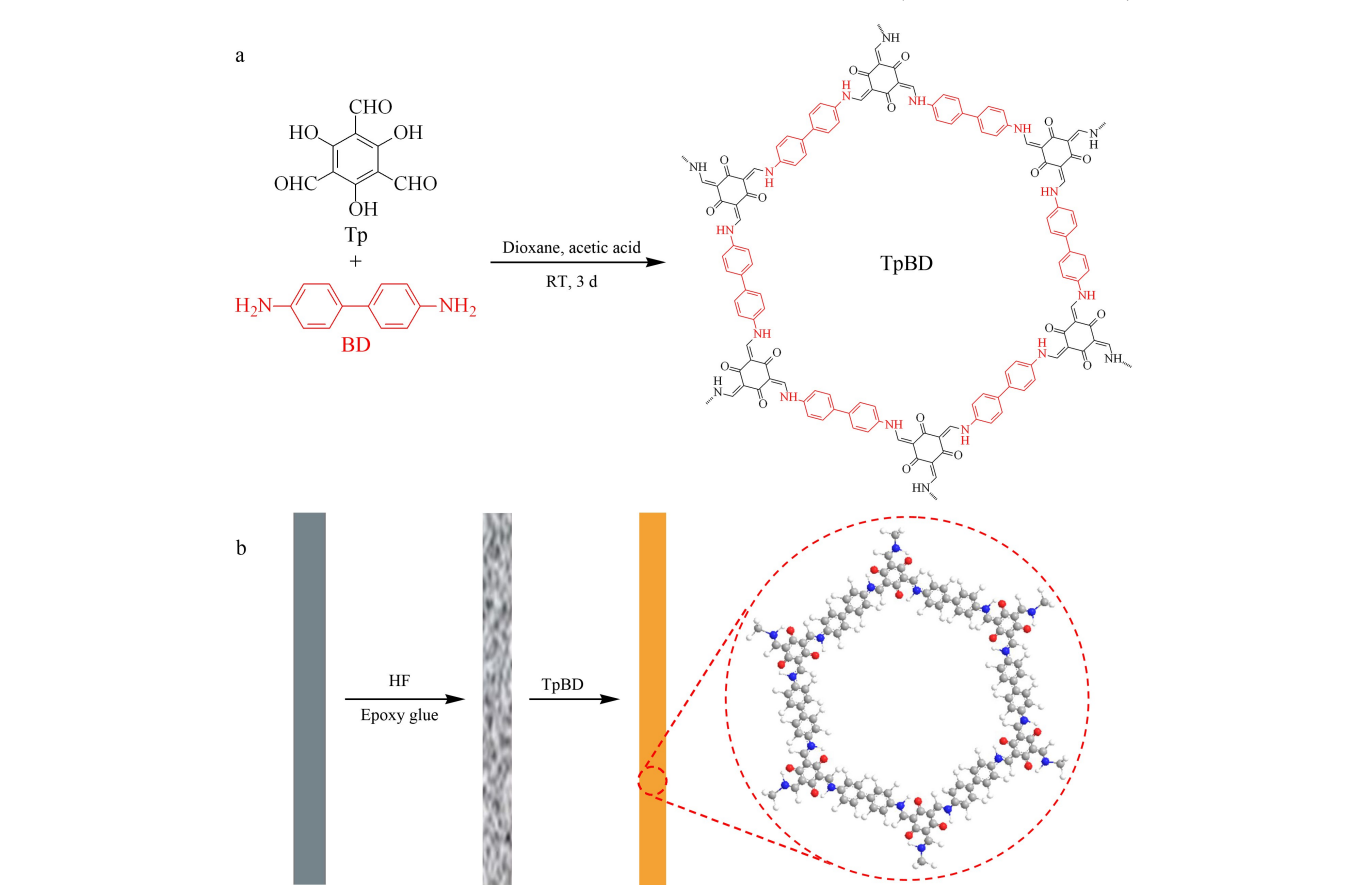
(a)室温溶液悬浮法合成TpBD和(b)制备TpBD-SPME纤维的示意图

不锈钢丝(20 cm×0.22 mm)前端3 cm部分浸入氢氟酸(HF)中刻蚀30 min,用去离子水超声清洗。刻蚀部分涂抹一层环氧树脂胶后垂直插入充满TpBD粉末的离心管中旋转几圈。该过程重复3次,获得所需厚度的纤维涂层(约9 μm),最后将涂层纤维在烤箱中120 ℃加热30 min。TpBD-SPME涂层纤维制备的过程见图1b。

### 1.4 样品前处理

茶样取自福建农林大学,处理方法参照国家食品安全标准(GB/T 23204-2008 4.5),并略有改进。5.0 g已粉碎茶样中加入15.0 mL乙腈,超声30 min后,离心5 min (5000 r/min),取上清液。残渣再经20.0 mL乙腈提取一次,合并两次提取液,旋转干燥后溶于2.0 mL丙酮,储存于4 ℃冰箱,备用。

### 1.5 固相微萃取

SPME萃取过程采用浸入式。取20.0 μL实际样品溶液,用水稀释成20.0 mL待测液,并置于25 mL血清瓶中,将TpBD纤维插入血清瓶中,在75 ℃具有磁力搅拌器的恒温水浴锅中萃取30 min。萃取结束后,纤维插入GC进样口,在280 ℃下解吸8 min。SPME纤维在两次萃取间隙,在290 ℃下老化30 min,以排除残留物质。

## 2 结果与讨论

### 2.1 SSA制备TpBD

通过考察不同反应时间下TpBD粉末X射线衍射(PXRD)和傅里叶变换红外(FT-IR)图谱,验证室温SSA是否能够合成良好结晶度的TpBD。如图2a所示,TpBD的结晶度随着反应时间的延长而增强,反应72 h时拥有良好的结晶度,这在3.3°和27.4°出现对应(100)和(001)面的特征峰得到证明,并且与前人报道^[16,17,18]^的相一致。从FT-IR图谱中可以看出,从反应30 min起C=C (1596 cm^-1^)和C-N (1289 cm^-1^)拉伸带已经出现,证实了配体发生反应,形成了*β*-酮烯胺结构(见图2b)。上述结果表明室温SSA 3 d就能合成TpBD。

**图 2:**
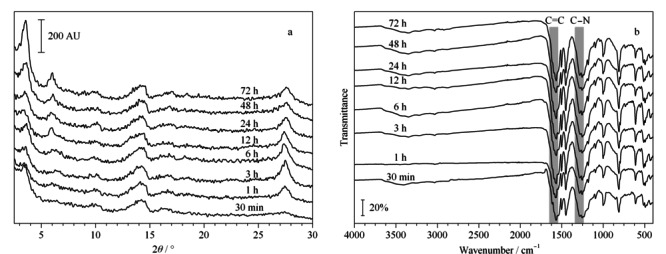
不同反应时间下TpBD的(a)PXRD和(b)FT-IR谱图

### 2.2 TpBD及其纤维的表征

为了考察TpBD的化学稳定性,对浸泡在水、有机溶剂和酸碱溶液中24 h后的TpBD进行PXRD(见图3a)和FT-IR(见图3b)测试。结果表明,TpBD在多种溶液中均能保持良好的晶型和化学结构。此外,TpBD的热稳定性高达350 ℃(见图3c)。经氮气吸附-脱附实验对TpBD的比表面积和孔隙率进行表征,TpBD的比表面积为230 m^2^/g,孔径尺寸为1.64 nm(见图3d)。较高的比表面、均一的孔道以及优异的热/化学稳定性使TpBD具备成为纤维涂层的条件。

**图 3:**
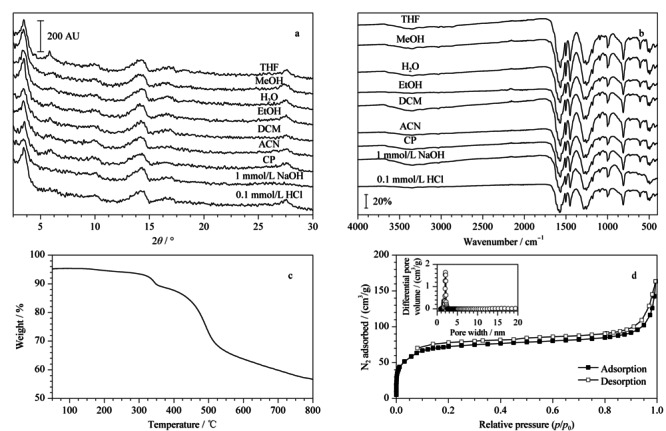
采用不同化学溶剂浸泡24 h后TpBD的(a)PXRD和(b)FT-IR谱图以及TpBD的 (c)热稳定性表征图和(d)等温氮气吸附-脱附曲线

通过涂层纤维的SEM图像可知(见图4),一层相对均匀的片状材料包覆在不锈钢丝表面,涂层厚度约为9 μm,其片状结构有利于目标物在涂层中的传质。

**图 4:**
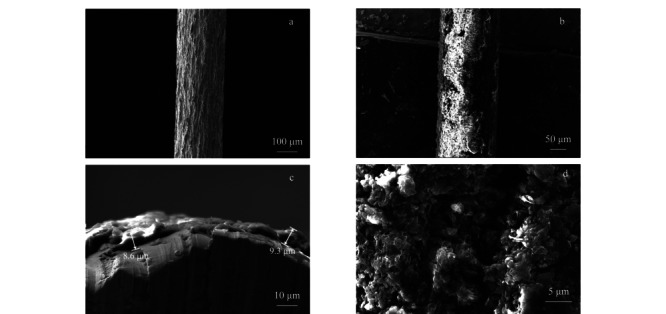
(a)刻蚀的不锈钢纤维、(b)TpBD涂层纤维、(c)TpBD纤维的横截面和(d)TpBD材料的SEM图

### 2.3 TpBD对PYs的SPME应用

萃取温度直接影响目标物在SPME纤维上的传质和吸附。本实验考察了萃取温度为65~85 ℃时,TpBD对PYs的萃取效率。由图5a可知,目标物的峰面积随着萃取温度而升高,在75 ℃时,达到最大值。但是随着后续温度的升高,萃取效率反而下降。这可能是因为PYs分子的运动速率随着萃取温度的提高而增加。同时,吸附是一个放热过程,过高的萃取温度会降低萃取效率。故选75 ℃为最佳萃取温度。

**图 5:**
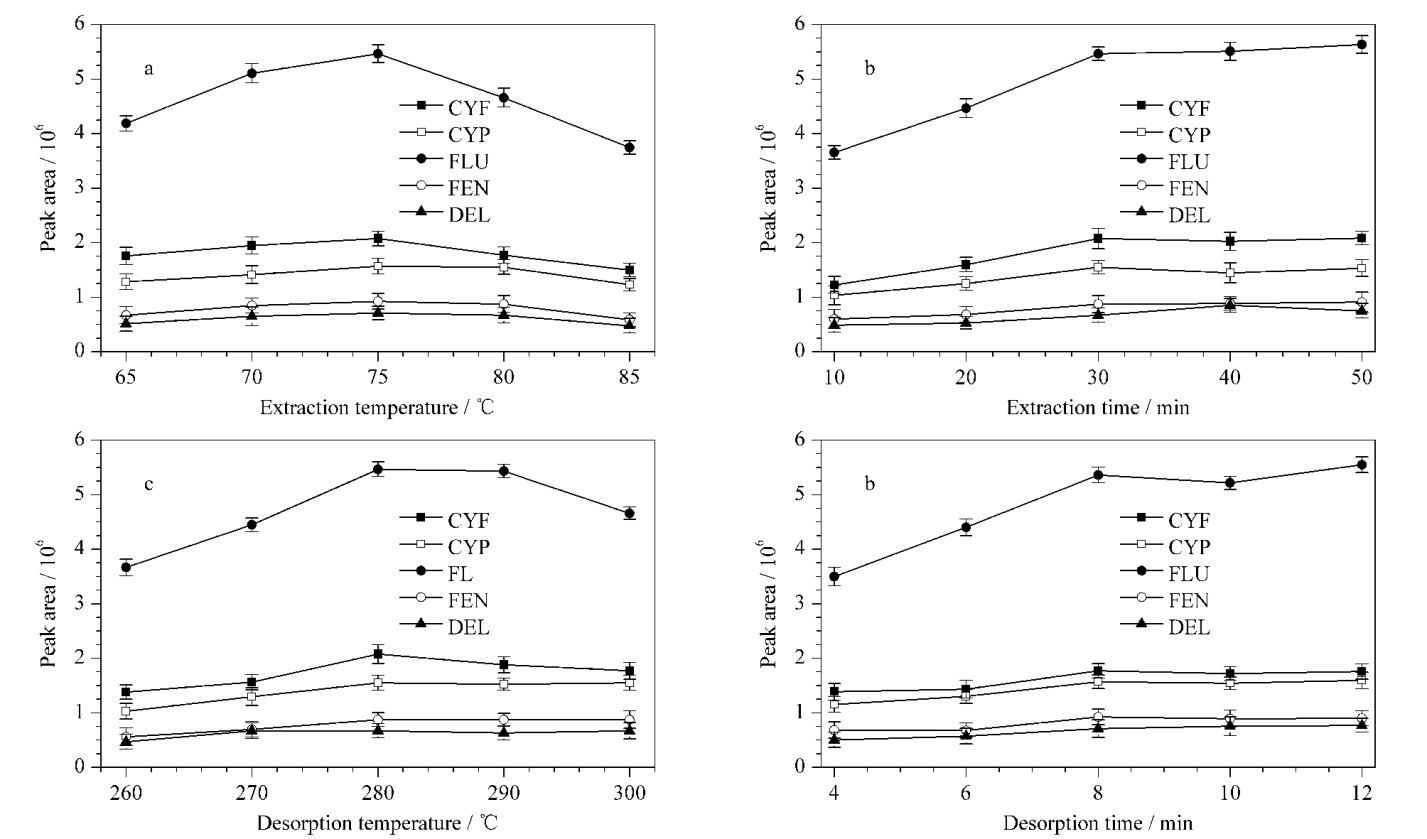
(a)萃取温度、(b)萃取时间、(c)解吸温度和(d)解吸时间对5种PYs(500 ng/L)萃取效率的影响(*n*=3)

SPME是基于吸附平衡的前处理方法,萃取效率与萃取时间呈正相关,直至达到萃取平衡。当萃取时间为10~50 min时,目标物的峰面积总体随着时间的增加而增加,直至30 min时,萃取达到平衡(见图5b)。故选30 min为最佳萃取时间。

为了最大程度将目标物从纤维上解吸下来,避免遗留效应影响后续纤维的使用,优化了解吸温度和解吸时间。从图5c和图5d可知,最佳的解吸温度和时间为280 ℃和8 min。

### 2.4 方法学考察

在最佳的SPME条件下,对建立的5种PYs分析方法进行评估。如表2所示,方法对5种PYs表现出良好的线性关系(相关系数(*R*)≥0.9991)、宽的线性范围(0.2~800 ng/L)和低的检出限(0.1~0.5 ng/L),对目标物萃取的富集倍数(EF)为702~2687(富集因子=目标物固相微萃取后标准曲线斜率/目标物不经任何处理直接进样所得的标准曲线斜率)。单根TpBD涂层纤维对500 ng/L的目标物溶液进行3次重复测定,以目标物的峰面积作为评判标准,其日内和日间相对标准偏差(RSD)分别为3.2%~5.5%和4.1%~8.7%。不同批次纤维间的RSD值为6.6%~11.0%。实验结果表明本方法具有良好的灵敏度、准确度和重复性。

**表 2:** 5种PYs的线性范围、相关系数、检出限、精密度和富集倍数

Analyte	Linear range/(ng/L)	*R*	LOD/ (ng/L)	RSD (*n*=3)/%			EF
				Single fiber		Fibers of batch-to-batch	
				Intra-day	Inter-day		
CYF	0.2-800	0.9995	0.1	5.5	4.9	8.9	1740
CYP	0.8-800	0.9997	0.3	4.3	4.1	6.6	1513
FLU	0.5-800	0.9995	0.1	5.5	5.4	8.7	1082
FEN	1-800	0.9998	0.3	3.2	5.4	11.0	2687
DEL	1-800	0.9991	0.5	3.9	8.7	9.6	702

### 2.5 与商业纤维和其他报道方法的比较

为了评估基于TpBD建立的PYs痕量检测方法的商业价值,对制备的纤维和CAR/PDMS商业纤维(75 μm)进行比较。实验结果表明,TpBD涂层纤维对PYs的萃取效果高于商业纤维。其中对FLU的萃取效率,TpBD涂层是普通的商业化萃取头的60倍。此外,本研究中,比较TpBD涂层纤维第1次和第120次对5种PYs的萃取效率,其效率并未出现显著差异,表明该纤维具有优异的使用寿命。

将本方法同文献方法进行对比(见表3),本方法具有更低的检出限和较宽的线性范围。这是因为PYs和TpBD之间的*π-π*相互作用、疏水效应和孔径选择性,使得TpBD对目标物具有突出的吸附能力。此外,COF较大的比表面积也有利于范德华力对拟除虫菊酯的吸附。

**表 3:** 本方法与其他文献方法的比较

Coating	Linear range/ (μg/L)	LOD/ (ng/L)		Analytical technique	Reference
MWCNTs	1-50	120-	165	GC-MS	[20]
MWCNTs/Ppy	1-10000	120-	430	GC-ECD	[21]
Hydrazone COF	1-1000	110-	230	GC-ECD	[15]
ZIF-90-NPC	0.3-50	100-	500	GC-μECD	[22]
TpBD	0.0002-0.8	0.1-	0.5	GC-MS/MS	this work

MWCNTs: multiwalled carbon nanotubes; Ppy: polypyrrole; ZIF: zeolitic imidazolate framework; NPC: nanoporous carbon; ECD: electron capture detector.

### 2.6 茶叶实际样的应用

将本方法用于绿茶和乌龙茶实际样中5种PYs的检测,并对该分析方法的回收率和精密度进行了考察。通过该方法,分别在绿茶和乌龙茶样品中检测出了CYF(0.4 ng/L和0.9 ng/L)、CYP(6.4 ng/L和1.7 ng/L)、FLU(2.1 ng/L和4.5 ng/L)、FEN(2.6 ng/L和2.6 ng/L)和DEL(2.7 ng/L和2.5 ng/L)的残留。此外,分别在茶叶空白样中进行加标回收试验,添加水平分别为5、20和100 ng/L,每个水平做3个平行样,回收率和精密数据见表4。5种PYs的平均回收率为80.2%~109.5%,并具有良好的重复性(RSD ≤9.5%)。图6是实际茶样和加标茶样(20 ng/L)的色谱图。结果表明本方法适用于检测茶产品中PYs残留。

**表 4:** 5种拟除虫菊酯类农药在绿茶和乌龙茶样品中的加标回收率及精密度(*n*=3)

Compound	Spiked level/ (ng/L)	Green tea			Oolong tea	
		Recovery/ %	RSD/ %		Recovery/ %	RSD/ %
CYF	5	92.7	8.5		82.2	8.0
	20	105.7	7.9		102.2	7.9
	100	99.6	7.8		91.4	2.9
CYP	5	81.8	9.5		88.0	9.1
	20	104.8	9.1		80.7	7.1
	100	89.8	8.2		93.5	1.2
FLU	5	94.8	7.9		105.9	5.9
	20	106.1	8.2		80.9	8.6
	100	88.1	3.0		86.5	3.2
FEN	5	92.1	7.3		89.4	5.4
	20	105.6	7.5		85.4	9.1
	100	94.7	3.2		91.4	4.2
DEL	5	109.5	6.7		107.7	7.5
	20	89.7	8.4		80.3	4.9
	100	81.4	5.3		88.3	7.8

**图 6:**
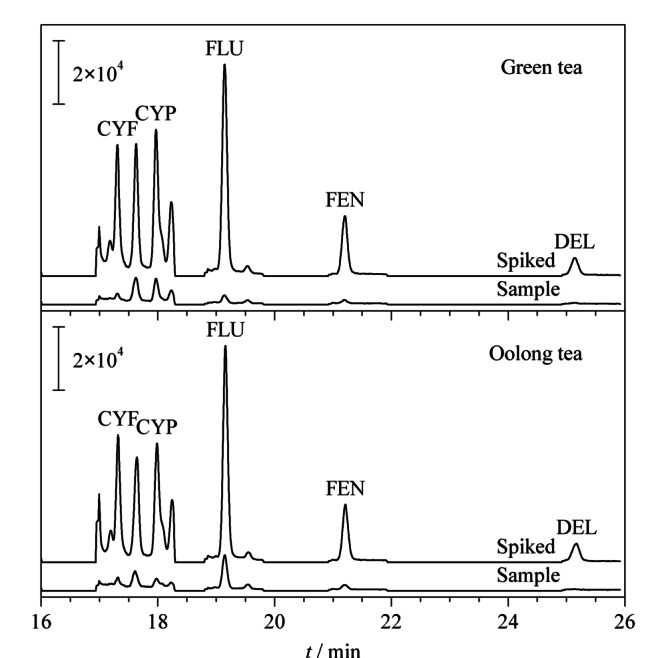
绿茶和乌龙茶样品中5种PYs(20 ng/L)的色谱图

## 3 结论

本实验通过合成步骤简单、反应条件温和的SSA合成具有优异热/化学稳定性、较高孔隙度和比表面的TpBD。将TpBD作为SPME涂层,与GC-MS/MS联用,建立测定茶叶中5种PYs农药残留的分析方法。方法具有令人满意的灵敏度、回收率和重复性,适用于绿茶和乌龙茶样品中PYs残留的检测。此外,通过SSA成功地制备了TpBD材料,证实了该方法具有较好的普适性,具有简便合成其他COFs材料的潜力。
